# All-cause excess mortality across 90 municipalities in Gujarat, India, during the COVID-19 pandemic (March 2020-April 2021)

**DOI:** 10.1371/journal.pgph.0000824

**Published:** 2022-08-16

**Authors:** Rolando J. Acosta, Biraj Patnaik, Caroline Buckee, Mathew V. Kiang, Rafael A. Irizarry, Satchit Balsari, Ayesha Mahmud

**Affiliations:** 1 Department of Biostatistics, Harvard T. H. Chan School of Public Health, Boston, Massachusetts, United States of America; 2 National Foundation for India, New Delhi, India; 3 Center for Communicable Disease Dynamics, Harvard T. H. Chan School of Public Health, Boston, Massachusetts, United States of America; 4 Department of Epidemiology, Harvard T. H. Chan School of Public Health, Boston, Massachusetts, United States of America; 5 Department of Epidemiology and Population Health, Stanford University School of Medicine, Palo Alto, California, United States of America; 6 Department of Data Science, Dana-Farber Cancer Institute, Boston, Massachusetts, United States of America; 7 Department of Emergency Medicine, Beth Israel Deaconess Medical Center, Harvard Medical School, Boston, Massachusetts, United States of America; 8 Department of Global Health and Population, Harvard T. H. Chan School of Public Health, Boston, Massachusetts, United States of America; 9 Department of Demography, University of California, Berkeley, California, United States of America; Universidade de São Paulo: Universidade de Sao Paulo, BRAZIL

## Abstract

Official COVID-19 mortality statistics are strongly influenced by local diagnostic capacity, strength of the healthcare and vital registration systems, and death certification criteria and capacity, often resulting in significant undercounting of COVID-19 attributable deaths. Excess mortality, which is defined as the increase in observed death counts compared to a baseline expectation, provides an alternate measure of the mortality shock—both direct and indirect—of the COVID-19 pandemic. Here, we use data from civil death registers from a convenience sample of 90 (of 162) municipalities across the state of Gujarat, India, to estimate the impact of the COVID-19 pandemic on all-cause mortality. Using a model fit to weekly data from January 2019 to February 2020, we estimated excess mortality over the course of the pandemic from March 2020 to April 2021. During this period, the official government data reported 10,098 deaths attributable to COVID-19 for the entire state of Gujarat. We estimated 21,300 [95% CI: 20, 700, 22, 000] excess deaths across these 90 municipalities in this period, representing a 44% [95% CI: 43%, 45%] increase over the expected baseline. The sharpest increase in deaths in our sample was observed in late April 2021, with an estimated 678% [95% CI: 649%, 707%] increase in mortality from expected counts. The 40 to 65 age group experienced the highest increase in mortality relative to the other age groups. We found substantial increases in mortality for males and females. Our excess mortality estimate for these 90 municipalities, representing approximately at least 8% of the population, based on the 2011 census, exceeds the official COVID-19 death count for the entire state of Gujarat, even before the delta wave of the pandemic in India peaked in May 2021. Prior studies have concluded that true pandemic-related mortality in India greatly exceeds official counts. This study, using data directly from the first point of official death registration data recording, provides incontrovertible evidence of the high excess mortality in Gujarat from March 2020 to April 2021.

## Introduction

On May 5, 2022, the World Health Organization (WHO) estimated the global excess mortality for 2020 and 2021, resulting directly or indirectly from the COVID-19 pandemic, to be between 13.3 to 16.6 million. Of these, 4.7 million deaths were attributed to those from India. The government of India to date attributes 481,000 deaths in the same period to COVID-19 [1, 2]. How governments report deaths from COVID-19 and compile national statistics however continues to vary widely around the world [3]. Most national statistics offices require COVID-19 to be listed as a cause of death on death certificates, for a death to be officially attributable to COVID-19 [4–6]. A well-functioning death registration system is then a prerequisite for these numbers to represent the true toll: patients with COVID-19 would need to have access to healthcare, health care providers would need to have the knowledge and clinical or laboratory tools necessary to diagnose COVID-19, and those recording deaths on death certificates would need to have the requisite training to note COVID-19 as an underlying cause of death [7, 8]. One or more of these conditions is often not met, resulting in underestimates of officially reported COVID-19 related deaths [9–11]. The undercounting in India has been attributed to limitations along several points in this pipeline. Even in states like Gujarat—the subject of our study here—where death registration may be relatively robust, the official death figures may not reflect the true toll of the pandemic because of limitations in access to care, limitations to diagnostic or confirmatory tests, and subsequently failure to record direct and indirect deaths due to COVID-19 on the death certificate.

In the absence of reliable death registration data in the aftermath of disasters and public health emergencies, scientists have relied on alternative methods to estimate deaths, including household based surveys, crematoria and funeral home body counts, or verbal autopsies [12–15]. In many countries, the estimation of all-cause mortality has provided an alternative proxy for the underestimation of COVID-19 attributable deaths in official statistics, whereby total observed deaths are compared to expected deaths computed from historical baselines [16–21]. These estimates include directly attributable deaths (those that died from SARS-Cov-2 and its complications) and indirectly attributable deaths (those that died from the indirect impacts of the pandemic, like disruptions in access to livelihoods, food security, public assistance, preventive health interventions, or medical care) [22, 23].

By December 2021, officials in the state of Gujarat in India stated that they had received up to 40,000 applications from families seeking compensation for COVID-19—a number that was nearly four times the then official count of 10,098 [24]. By April 2021, the Gujarat High Court had expressed concern that the death counts noted by the government seemed to be underestimates, observing that the “suppression and concealment of accurate data would generate more serious problems including fear, loss of trust, and panic amongst the public at large” [25].

To understand whether there is a gap between the official and true toll as suggested by modeling estimates, and to quantify this gap, we calculated excess mortality by directly counting all deaths recorded in death registers in a non-random sample of municipalities in the state of Gujarat. Here, we use deaths data from 90 municipalities in the State of Gujarat in India to examine the impact of the COVID-19 pandemic on all-cause mortality. We explore the differences in mortality by age and sex over the course of the pandemic, and estimate the excess deaths resulting from the pandemic.

## Methods

We used de-identified data from civil death registers, the official records at the municipal jurisdictional level. In India, deaths are first recorded in the death registers maintained at the Gram Panchayats (village committees) in rural India, and in municipalities and municipal corporations in urban India, typically within a few weeks of the death. The data are then aggregated at the district level and submitted to the national Civil Registration System (CRS) [26, 27]. It may take up to nine months from the end of the financial year for the CRS to be fully updated and validated by the government of India. Therefore, data collated directly from the death registers and maintained locally are the most comprehensive source of deaths from 2020 and 2021 currently available—until the CRS is fully updated. The data comprise a convenience sample of 90 municipalities, made publicly available by The Reporter’s Collective (RC). The RC, a group of investigative journalists [28, 29], filed a request in May 2021, through India’s Right to Information Act, for death registration data from 90 of 162 total municipalities in Gujarat, for 2019, 2020, and for all months in 2021 up to the date of filing in May. Photocopies of the death registers were sent to the RC. The data were tabulated and translated to English, and manually scanned for typographic or date formatting errors. Importantly, these data, with the exception of Gandhinagar, only include the urban population of the municipalities.

We conducted an analysis of these data directly derived from death registers from the 90 municipalities in the state of Gujarat, representing a population of at least 4.9 million (according to the 2011 census), or approximately 8% of the total state population of 60.4 million [30]. These data encompass all recorded deaths from January 2019 to May 2021, and include date of death, date of registration, gender, age, and place of death information. We do not have access to data after May 2021, as no subsequent request was filed by the RC nor any other entity to the best of our knowledge. This study used deidentified and publicly available data on deceased individuals, and therefore it was IRB exempt.

In Gujarat, according to the National Family Health Survey (NFHS) 2019–20, 93% of all deaths “of usual residents of households” in Gujarat are recorded in the civil death registers. Death registration completeness reaches nearly 96% in urban areas, 92% in rural areas, 94% for males, and 91% for females, overall [31]. While the attribution of COVID-19 as a cause of death may be limited on the death certificates, this high level of completeness supports reliable estimation of all-cause excess mortality. Using these records, we computed weekly mortality counts for males and females, and by four age groups: less than 20, 20 to 40, 40 to 65, and 65 years and over, from January 2019 to April 2021. To minimize possible reporting lags, we only use data until April 2021 (S1 Fig).

### Statistical analysis

Based on the reported COVID-19 case and mortality data in India from early 2020, we considered data from January 2019, to February 2020 to represent baseline mortality. We compared the baseline mortality data to observed counts from March 2020 onwards to estimate excess mortality. Due to the paucity of data, ascertaining excess mortality by both age and sex interactions is not possible. As an alternative, we conducted three analyses. In the first, we aggregated data across all demographic indicators for each municipality, thus we had weekly death counts per jurisdiction. For the second and third analyses, we aggregated data by gender and the age groups mentioned above, respectively. We refer to the three analyses as the *overall* analysis, the *gender* analysis, and the *age* analysis.

To estimate expected mortality over the course of the pandemic we, assume that *Y*_*tmd*_ ∼ *Poisson*(*μ*_*tmd*_), where *Y*_*tmd*_ is the number of deaths at week *t* in municipality *m* for the demographic group *d*. Note that the index *d* is ignored in the marginal analysis. The mean of the distribution is modeled as

$$\begin{array}{c} \mu_{tmd}=\text{exp}\;\{\gamma_{d}+\gamma_{md}+s_{d}\left(w_{t}\right)\}, \end{array}$$ (1)

for *t* = 1, …, *T*, *m* = 1, …, *M*, and *d* = 1, …, *D*. In model 1, *μ*_*tmd*_ represents the average number of deaths at week *t* in municipality *m* for demographic group *d*, *γ*_*d*_ is a demographic-specific intercept that represents the average number of deaths per week across all municipalities, *γ*_*md*_ is a municipality-specific intercept that represent deviations in the average number of deaths per week in municipality *m* from *γ*_*d*_, *s* is a harmonic component that accounts for seasonality, *w*_*t*_ ∈ {1, …, 52} corresponds to the week of the year associated with *t*, *T* is the number of observations in the time-series, *M* is the number of municipalities, and *D* is the number of demographic groups. Population estimates for 2019–2021 are unavailable as the last census was conducted in 2011, and population projections for these years are not available at the municipality level. Jurisdictional boundaries have also changed since the last census for several municipalities, making it challenging to use data from the 2011 census. We therefore assumed that the population size in each municipality was constant from 2019 to 2021, and did not include a population offset in our mean model. We used data from January 2019 to February 2020 to fit model 1 via maximum likelihood assuming an overdispersed Poisson distribution. Then, we estimated the expected mortality for each municipality from March 2020 to April 2021 using the estimated model parameters. To account for variation in the observed counts during the period of interest, let *t*′ be a week after the onset of Covid-19 with corresponding outcome *Y*_*t*′*md*_ ∼ *Poisson*(*μ*_*t*′*md*_). For each municipality, we fit the following model:

$$\begin{array}{c} \lambda_{t^{\prime }md}={\hat{\mu }}_{t^{\prime }md}\;\text{exp}\;\{f_{md}\left(t^{\prime }\right)\}, \end{array}$$ (2)

with λ_*t*′*md*_ the average number of deaths at *t*′, *f*_*md*_ a smooth function of time that represents deviations from expected mortality based on historical data, and ${\hat{\mu }}_{t^{\prime }md}$ an offset representing expected mortality at *t*′. We modeled *f*_*md*_ with a natural cubic spline where the number of internal nodes depended on data quality. As before, we fit model 2 via maximum likelihood assuming an overdispersed Poisson distribution. We estimated excess deaths at *t*′ with:

$$\begin{array}{c} {\hat{\Delta }}_{t^{\prime }md}={\hat{\lambda }}_{t^{\prime }md}-{\hat{\mu }}_{t^{\prime }md}, \end{array}$$ (3)

with variance

$$\begin{array}{c} \text{Var}\;({\hat{\Delta }}_{t^{\prime }md})={\hat{\phi }}_{md}{\hat{\lambda }}_{t^{\prime }md}-\text{Var}\;({\hat{\mu }}_{t^{\prime }md}), \end{array}$$ (4)

where ${\hat{\phi }}_{md}$ is the estimated dispersion parameter from model 2. We estimated cumulative excess death and associated confidence intervals by summing the excess death estimates and corresponding variance estimates, respectively. Finally, we computed municipality-specific percent changes from average mortality as a function of time, and amalgamated these metrics to ascertain deviations in mortality from expectation for all municipalities combined [21]. In the Results section, we round our excess death estimates proportional to the standard error. For example, if the standard error is in the hundreds, we round our estimate to the nearest tens.

For each analysis, we removed municipalities due to missing data. Specifically, we excluded municipalities with at least 60% missing data in either the train (January 2019 to February 2020) or test (March 2020 to April 2021) period. This resulted in the exclusion of the two municipalities of Jetpur and Modasa (from a total of 92) for all three analyses (S2 Fig). Our final sample, thus, includes 90 municipalities in total. For the gender analysis, we considered missing data in the male and female categories (due to coding inconsistencies across municipalities), and further removed 13 municipalities using the same exclusion criteria (S3 Fig). Finally, for the age analysis, we categorized the data into four age groups: less than 20 years, 20 to 40, 40 to 65, and 65 and over. For each age group, we removed municipalities that did not comply with the exclusion criteria (S1 Table). The data and code to reproduce our analyses are available at: https://github.com/RJNunez/gujarat-ed-study.

## Results

### Excess deaths overall

**Fig 1:**
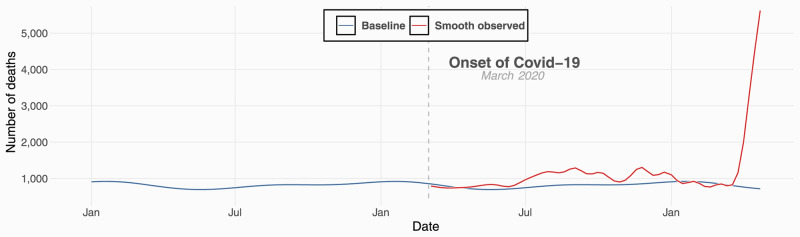
Model fit for weekly death counts. Model fit for weekly death counts amalgamated from multiple municipalities in Gujarat, India. The gray data points are weekly death counts, the dashed-vertical line represents the onset of Covid-19, the blue curve represents the expected weekly death counts based on historical data, and the red curve represents the smooth observed weekly death counts during Covid-19.

Across these 90 non-representative municipalities, 77,781 total deaths were recorded over the course of the pandemic, from March 2020 onwards. While deaths were higher in both 2020 (50,866) and 2021 (26,915 up to April) compared to 2019 (42,246), the sharpest increase in deaths was observed during the period encompassing the start of the second wave of the pandemic in March and April 2021 (Fig 1). From our overall analysis, we estimated 21,300 excess deaths [95% CI: 20, 700, 22, 000] since March 2020, representing a 44% [95% CI: 43%, 45%] increase over the expected baseline, most of which occurred at the start of the second wave in March 2021 (S4 Fig).

### Percent change in mortality overall, by age, and by sex

**Fig 2:**
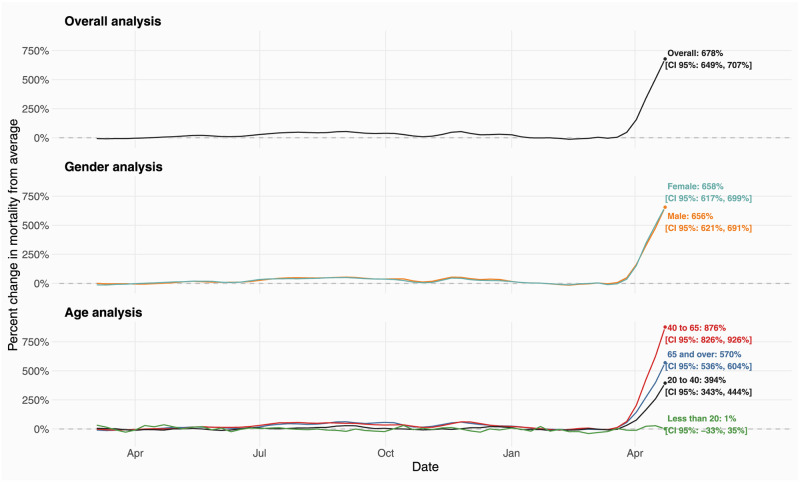
Estimated percent change in mortality from average in Gujarat, India, from March 2020 to April 2021. Estimated percent change in mortality from average in Gujarat, India, from March 2020 to April 2021. The solid-curves represent percent changes from average mortality for each group. 95% confidence intervals were omitted for better readability. The point estimate and corresponding 95% confidence intervals for April 16, 2021, the week of peak excess mortality, are displayed in text on the right and highlighted with a data point.

During the first wave of the pandemic, all-cause mortality started to increase in May 2020 and continued to exceed the expected baseline until January 2021 (Fig 2). Over this time period, we estimated a 30.5% [95% CI: 29.4%, 31.6%] increase in mortality from our overall analysis, a 32.2% [95% CI: 30.9%, 33.6%] and 28.2% [95% CI: 26.7%, 29.8%] increase for males and females, respectively, and a 33.7% [95% CI: 32.1%, 35.4%] and 34.1% [95% CI: 32.6%, 35.5%] increase for the 40 to 65 and the 65 years and over cohort, respectively. The 20 to 40 years age group experienced a 5.46% [95% CI: 3.10%, 7.82%] increase and we found no evidence of excess mortality for the youngest group during the first wave.

Starting from March 2021, we once again observed an increase in mortality —this time precipitous —that varied by age but not by sex. For this period, we found an overall increase in mortality of 339% [95% CI: 330%, 347%]. For males and females, we estimated increases of 327% [95% CI: 317%, 337%] and 334% [95% CI: 322%, 346%], respectively. For the groups, 20 to 40, the 40 to 65, and the 65 years and over, we found increases of 196% [95% CI: 180%, 211%], 438% [95% CI: 423%, 452%], and 284% [95% CI: 275%, 294%], respectively. In our sample, peak mortality occurred in the last week of available data, where the steep positive slope in Fig 2 suggests that excess mortality very likely kept increasing in the subsequent weeks. In this week, we estimated a 678% [95% CI: 649%, 707%] increase in overall mortality. We further found a 658% [95% CI: 617%, 699%] and a 656% [95% CI: 621%, 691%] increase in mortality for females and males, respectively. Conversely, we found substantial variation across age groups. We found no evidence of inordinate mortality for those 20 years and younger. For the 20 to 40 years age group, we found an increase of 394% [95% CI: 343%, 444%]. Of note, the 40 to 65 years age group experienced excess mortality larger than the 65 and over age group. For the former, we estimated an increase of 876% [95% CI: 826%, 926%] and for the latter an increase of 570% [95% CI: 536%, 604%].

### Excess mortality by age and by sex for municipalities

**Fig 3:**
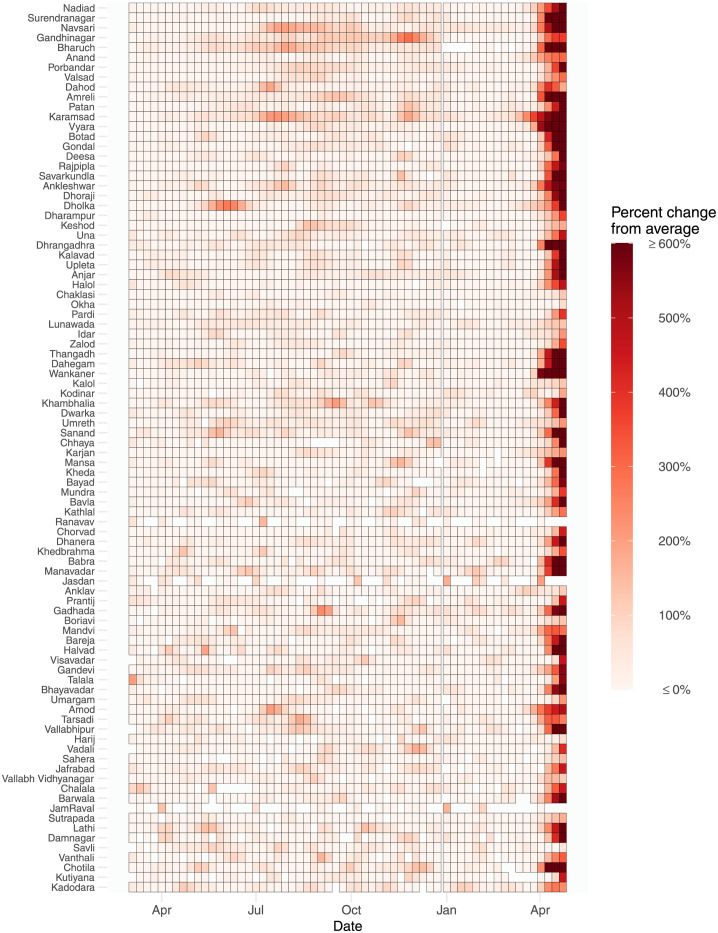
Estimated percent increase in mortality in the 90 municipalities, March 2020 to April 2021, from our marginal analysis. Estimated percent increase in mortality in the 90 municipalities, March 2020 to April 2021, from our marginal analysis.

Across all 90 municipalities, the largest increases in mortality occurred in the second wave of the pandemic (Fig 3). In the last week of available data, 85 of the 90 municipalities we studied experienced increases in mortality over 100%. Forty-seven of the 90 municipalities experienced increases in mortality over 500% during the same week. Excess mortality differed by age and sex at the municipal level (S5 & S6 Figs). We found this to be particularly true in larger municipalities, where we appraised the size of each jurisdiction via the estimated intercepts in model 1 (S7 Fig). We further found that over 80% of the municipalities experienced increases in mortality over 100% in all demographic groups except the two youngest age cohorts. Lastly, in over 50% of the municipalities, the 40 to 65 years groups experienced an increase of over 500%.

## Discussion

We describe mortality trends across 90 municipalities in Gujarat, India over the course of the COVID-19 pandemic, until April 2021. The official Covid-19 death count for the *entire* state of Gujarat is 10,098 [32]. However, in a recent hearing to India’s Supreme Court, Gujarat’s government accepted almost 10,000 more Covid-19 deaths [33]. Our results suggest that in these 90 municipalities alone there were 21,300 [95% CI: 20, 700, 22, 000] excess deaths from March 2020 to April 2021, far exceeding the official count. The 40 to 65 age group experienced the greatest increase from baseline mortality relative to other age groups, and both males and females experienced similar increases in mortality. The vast majority of these excess deaths likely represent direct deaths from COVID-19, in the absence of any other known catastrophe. A small percentage of these would include deaths from the indirect impact of the pandemic, and from causes unrelated to the pandemic.

Our study has several limitations. First, the data only represent at least 8% of the population of Gujarat, based on the 2011 census. Given the change in jurisdictional boundaries between the 2011 census and their status in 2021, we were able to assess the population size for only 63 municipalities, using data from the 2011 census. Our sample is therefore likely representative of a larger percentage of the state’s population. These data represent urban populations. With the exception of Gandhinagar, they include neither the municipal corporations of other large urban centers, nor the rural gram panchayats. As the 2021 census was not conducted [34, 35], socioeconomic data for these municipalities is currently unavailable. Hence it is not possible to assess the relationship between excess mortality and multiple social indicators. Though the municipalities were spread across the state, they represent a convenience sample rather than a random sample. We are therefore unable to extrapolate our results to estimate deaths across the entire state. Note, however, that our cumulative excess deaths estimate, using only 90 out 162 municipalities in the state, doubles the government official COVID-19 death toll for the *entire* state. Our estimate is also a lower bound for the death toll of the pandemic in 2021, given that the large Delta wave of the outbreak peaked after the period for which data were available. According to the NFHS, a high percentage of deaths are actually recorded in the death registers, rendering our analysis highly representative of mortality in the examined municipalities. The strikingly high mortality is also consistent with media reports and lived experience and likely representative of the general trend across the state. Our results cannot be extrapolated to estimate excess mortality nationally, given limitations with their representativeness and the socioeconomic and health system heterogeneity across India. A study of data from health facilities across several Indian states found that Gujarat had the highest increase in mortality during the pandemic [36].

Second, since the data on the yearly population size for each municipality are not available, for a variety of reasons that include changing jurisdictional boundaries, we were unable to calculate mortality rates and make comparisons across municipalities. For the same reason, we were unable to assess excess mortality rates by demographic indicators. The last published census data are from 2011 [30]. While data from electoral rolls are more recent, they do not map to the same geospatial unit of the municipality, and cannot be easily used. We therefore make the assumption that the population remained unchanged between January 2019 and April 2021. Our results will be biased if there was significant migration in or out of the state between our baseline period and the pandemic period. Had population sizes significantly increased (or decreased) over time, our excess mortality estimate would be an overestimate (or underestimate). Because mortality varies strongly with age, both for COVID-19 and all-cause mortality, an ideal comparison would also adjust for age-specific population changes.

Third, we only have baseline (pre-pandemic) data from January 2019 to February 2020. Since the baseline period for fitting the model is relatively short, we may not be sufficiently capturing year-to-year variations in mortality. However, the sharp increase in mortality observed in 2021 is unlikely to fall within the bounds of normal yearly variability in mortality. Finally, there may be lags in recording of deaths in the death registry, and not all deaths may yet be registered. This might have been exacerbated by the implementations of lockdowns [37, 38]. Given that death registration is a requisite for several subsequent services and legal processes that the family would encounter, we proceed with the assumption that deaths were subsequently registered, even if delayed by the lockdowns. We only used data until April 2021, to avoid including incomplete data from possible reporting lags. According to media reports, mortality continued to be high, or rose, in May 2021, and is not yet included in the published data or in our estimates.

Despite these limitations, the rapid increase in all-cause mortality, especially during the second wave of the pandemic, is irrefutably high. We estimated a 678% increase [95% CI: 649%, 707%] in deaths in the last week of available data, in April 2021, in the municipalities studied. Our results suggest that April 2021 was the beginning of the second wave, a finding that is in agreement with recent estimates [36, 39, 40]. This increase is among the highest percentage increase in deaths recorded anywhere in the world. In April 2020, Ecuador recorded a 411% increase; in April 2021, Peru recorded a 345% increase [41]. This large discrepancy between official COVID-19 death counts and excess mortality underscores the need to rectify how official death counts are collated. Reliance on death certificates as the single source of truth is sub-optimal, anywhere in the world, when access to health systems, testing availability, and death certification accuracy and completeness are weak.

The high mortality counts across age groups, and especially in the 40–65 years group, warrants further investigation into the impact of underlying social determinants and the efficacy of clinical protocols and public health policies on mortality. The lack of relevant data precludes these necessary analyses. Globally, data on population estimates, testing, and clinical outcomes, where available, have facilitated contextually intelligent public health planning and response. State supported data transparency and availability can in fact help local scientists focus on knowledge generation, and provide citizens and the state the tools needed to strengthen health systems.

## Supporting information

S1 Fig Weekly death counts amalgamated from the 90 municipalities in Gujarat, India. (PDF)

S2 Fig Weekly death counts for Jetpur and Modasa. (PDF)

S3 Fig Weekly death counts for municipalities not included in the gender analysis. (PDF)

S4 Fig Cumulative excess deaths from March 2020 to April 2021 for all 90 municipalities combined. (PDF)

S5 Fig Estimated percent increase in mortality in the 90 municipalities during the COVID-19 pandemic from our gender analysis. (PDF)

S6 Fig Estimated percent increase in mortality in the 90 municipalities during the COVID-19 pandemic from our age analysis. (PDF)

S7 Fig Estimated municipality-specific intercepts from model 1 from our marginal analysis. (PDF)

S1 Table Municipalities included and not included in each analysis. (CSV)
