# Supplementary figures and images for: All-cause excess mortality across 90 municipalities in Gujarat, India, during the COVID-19 pandemic (March 2020-April 2021)
Source: PLOS Glob Public Health. 2022 Aug 16;2(8):e0000824. doi: 10.1371/journal.pgph.0000824 (PMC10021770; doi:10.1371/journal.pgph.0000824)

Number of deaths

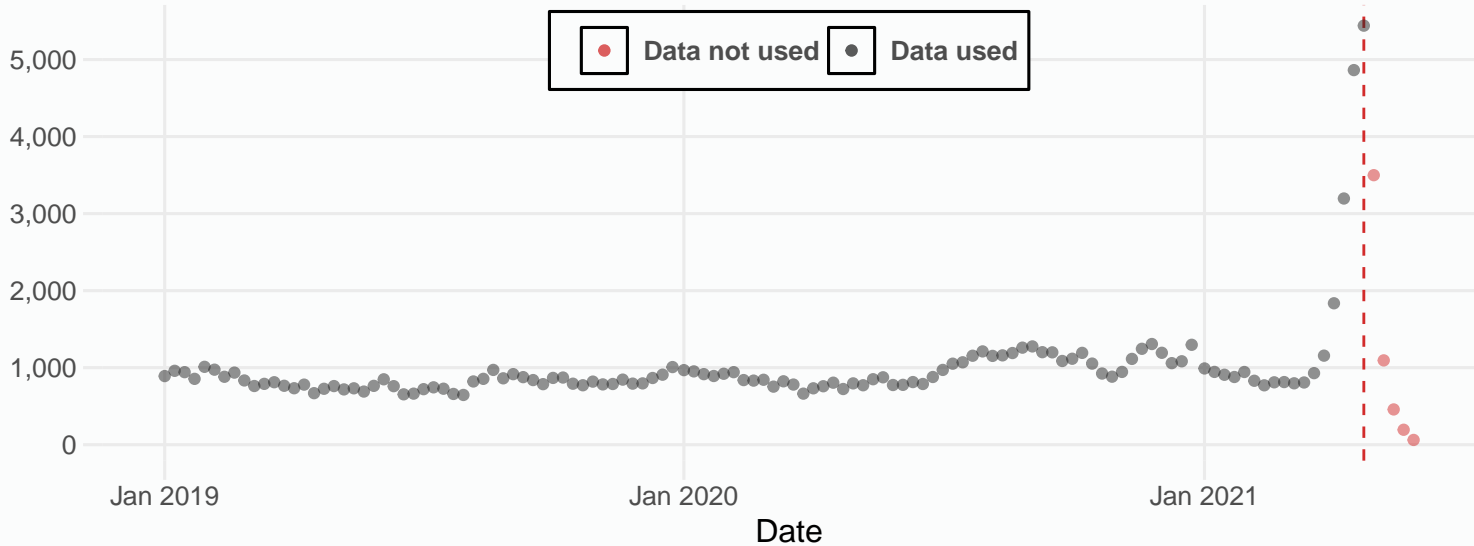

Supplement: S1 Fig — (PDF) [file pgph.0000824.s001.pdf]

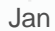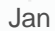

Supplement: S2 Fig — (PDF) [file pgph.0000824.s002.pdf]

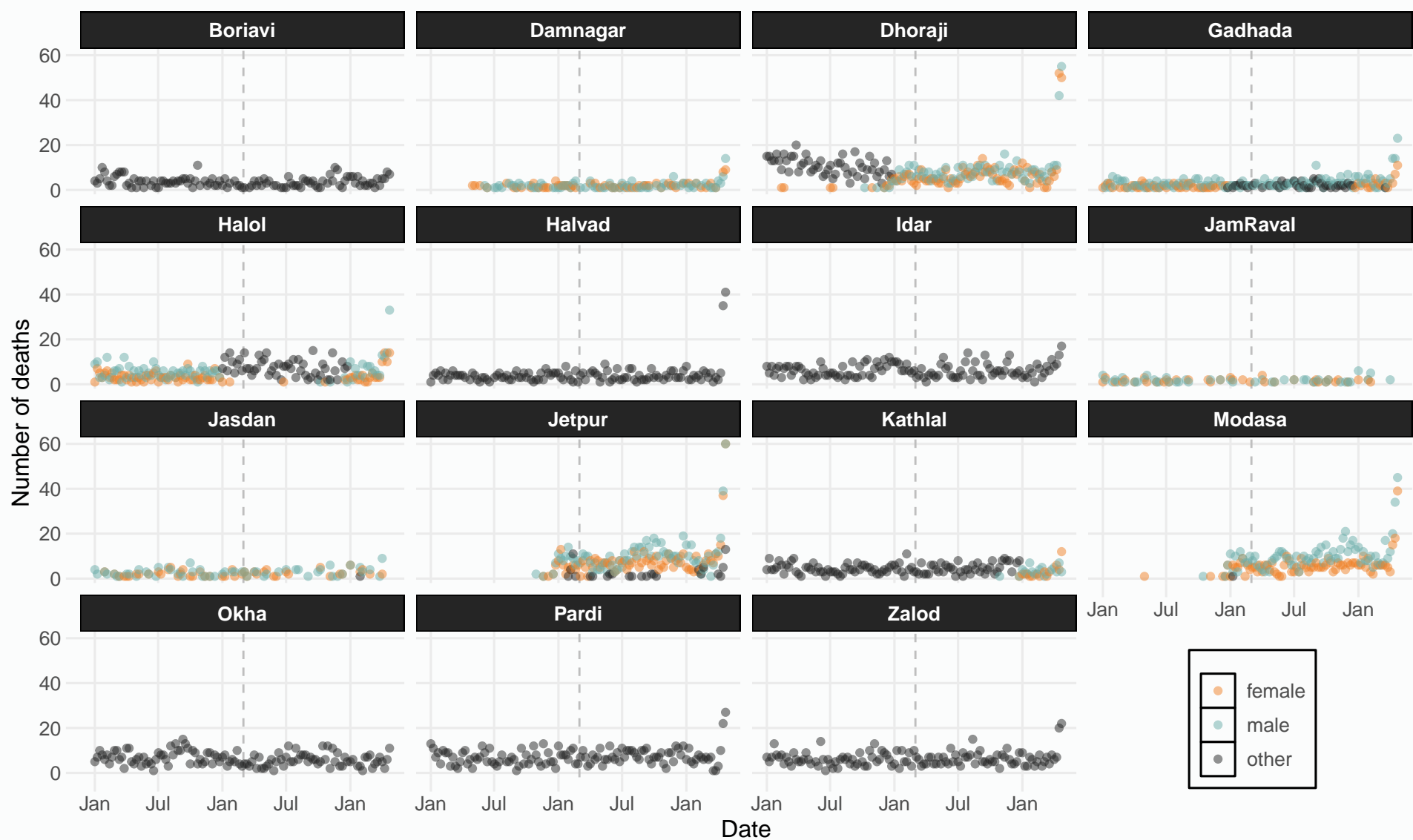

Supplement: S3 Fig — (PDF) [file pgph.0000824.s003.pdf]

Cumulative excess deaths

**21,300 [95% CI: 20,700 to 22,000]**

20,000

15,000

10,000

5,000

0

Apr

Jul

Oct

Jan

Apr

Date

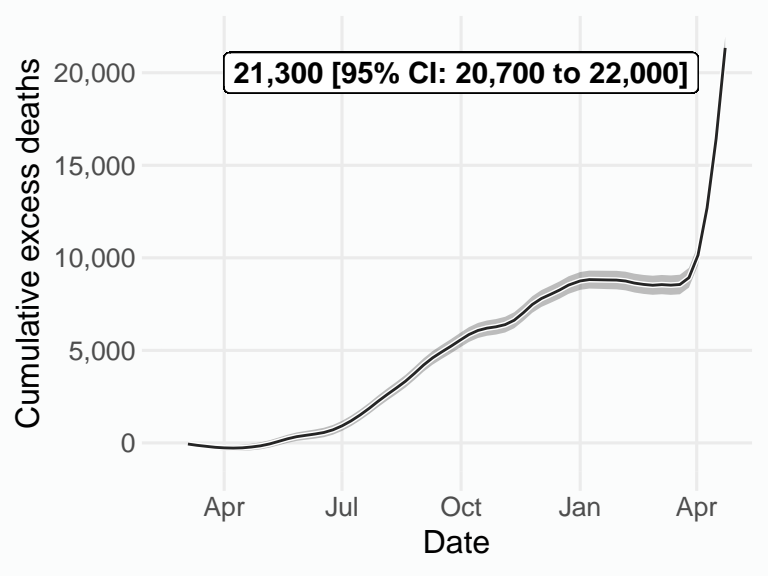

Supplement: S4 Fig — (PDF) [file pgph.0000824.s004.pdf]

# Female

# Male

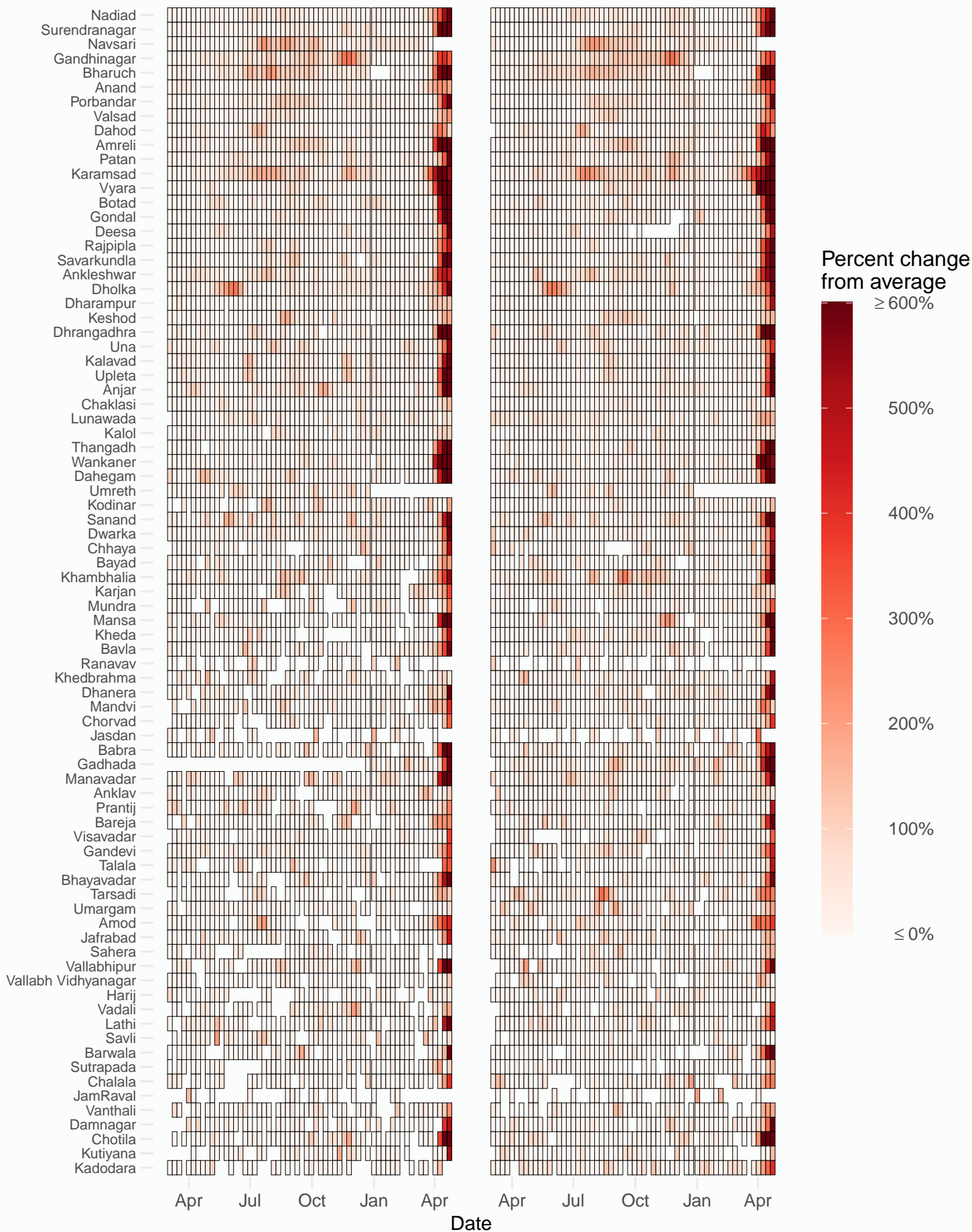

Supplement: S5 Fig — (PDF) [file pgph.0000824.s005.pdf]

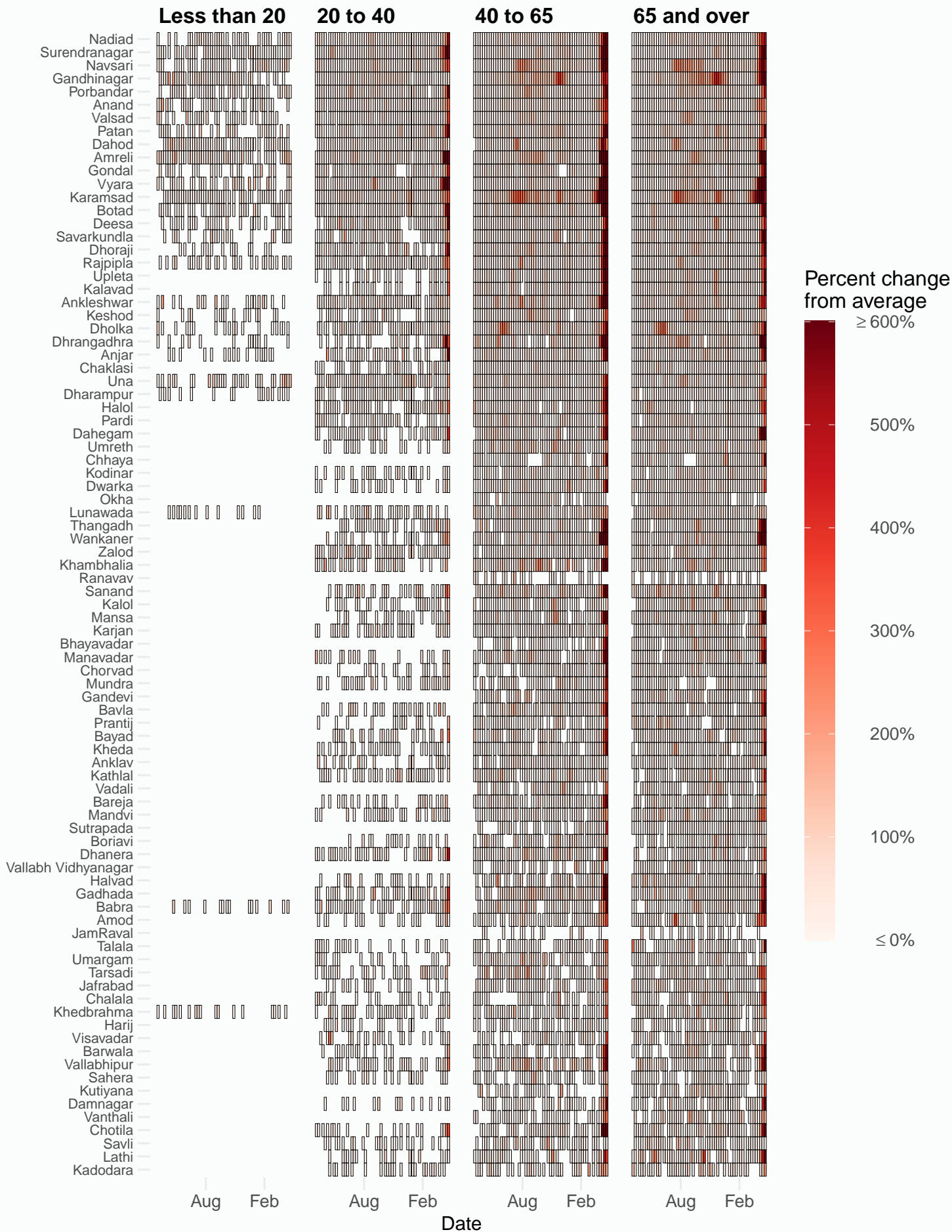

Supplement: S6 Fig — (PDF) [file pgph.0000824.s006.pdf]

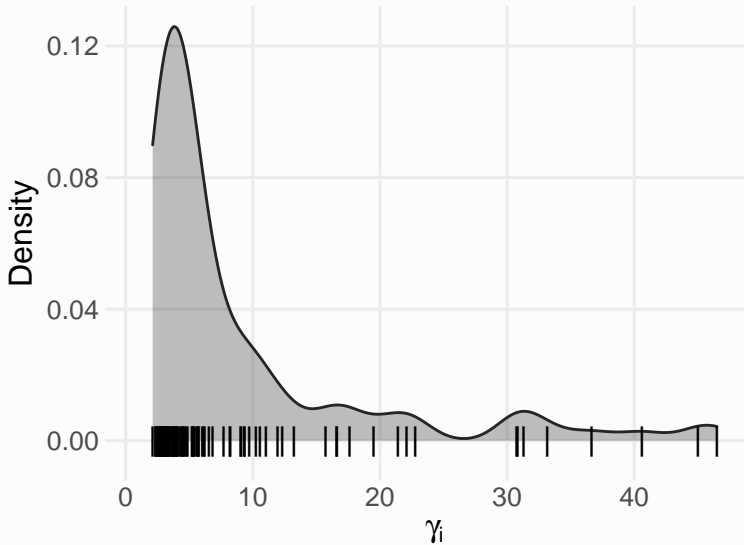

Supplement: S7 Fig — (PDF) [file pgph.0000824.s007.pdf]
